# AAV-Mediated Gene Supplementation Therapy in Achromatopsia Type 2: Preclinical Data on Therapeutic Time Window and Long-Term Effects

**DOI:** 10.3389/fnins.2017.00292

**Published:** 2017-05-24

**Authors:** Regine Mühlfriedel, Naoyuki Tanimoto, Christian Schön, Vithiyanjali Sothilingam, Marina Garcia Garrido, Susanne C. Beck, Gesine Huber, Martin Biel, Mathias W. Seeliger, Stylianos Michalakis

**Affiliations:** ^1^Division of Ocular Neurodegeneration, Centre for Ophthalmology, Institute for Ophthalmic Research, Eberhard Karls-Universität TübingenTuebingen, Germany; ^2^Department of Pharmacy, Center for Drug Research, Center for Integrated Protein Science Munich, Ludwig-Maximilians-Universität MünchenMunich, Germany

**Keywords:** AAV vector, gene therapy, CNGA3, achromatopsia, subretinal delivery, cone function restoration, non-invasive diagnostic techniques, photoreceptor cells

## Abstract

Achromatopsia type 2 (ACHM2) is a severe, inherited eye disease caused by mutations in the *CNGA3* gene encoding the α subunit of the cone photoreceptor cyclic nucleotide-gated (CNG) channel. Patients suffer from strongly impaired daylight vision, photophobia, nystagmus, and lack of color discrimination. We have previously shown in the *Cnga*3 knockout (KO) mouse model of ACHM2 that gene supplementation therapy is effective in rescuing cone function and morphology and delaying cone degeneration. In our preclinical approach, we use recombinant adeno-associated virus (AAV) vector-mediated gene transfer to express the murine *Cnga3* gene under control of the mouse blue opsin promoter. Here, we provide novel data on the efficiency and permanence of such gene supplementation therapy in *Cnga3* KO mice. Specifically, we compare the influence of two different AAV vector capsids, AAV2/5 (Y719F) and AAV2/8 (Y733F), on restoration of cone function, and assess the effect of age at time of treatment on the long-term outcome. The evaluation included *in vivo* analysis of retinal function using electroretinography (ERG) and immunohistochemical analysis of vector-driven *Cnga3* transgene expression. We found that both vector capsid serotypes led to a comparable rescue of cone function over the observation period between 4 weeks and 3 months post treatment. In addition, a clear therapeutic effect was present in mice treated at 2 weeks of age as well as in mice treated at 3 months of age at the first assessment at 4 weeks after treatment. Importantly, the effect extended in both cases over the entire observation period of 12 months post treatment. However, the average ERG amplitude levels differed between the two groups, suggesting a role of the absolute age, or possibly, the associated state of the degeneration, on the achievable outcome. In summary, we found that the therapeutic time window of opportunity for AAV-mediated *Cnga3* gene supplementation therapy in the *Cnga3* KO mouse model extends at least to an age of 3 months, but is presumably limited by the condition, number and topographical distribution of remaining cones at the time of treatment. No impact of the choice of capsid on the therapeutic success was detected.

## Introduction

Achromatopsia (ACHM) is an autosomal recessively inherited disorder affecting cone-mediated vision with an overall prevalence of 1:30,000 (Poloschek and Kohl, 2010; Zobor et al., 2015; Aboshiha et al., 2016). Patients with ACHM suffer from severely impaired daylight vision, poor visual acuity, photophobia, nystagmus, and lack of the ability to discriminate colors (Biel and Michalakis, 2007). In addition to these functional defects, ACHM patients present varying degrees of cone photoreceptor degeneration. Currently six genes have been linked to ACHM: *ATF6* (activating transcription factor 6A; MIM 605537) (Kohl et al., 2015), *CNGA3* (cyclic nucleotide–gated cation channel α3; MIM 600053; ACHM2, MIM 216900) (Kohl et al., 1998), *CNGB3* (cyclic nucleotide–gated cation channel β3, MIM 605080; ACHM3, MIM 262300) (Kohl et al., 2000; Sundin et al., 2000), *GNAT2* (guanine nucleotide–binding protein G(t) subunit α2; ACHM4, MIM 139340) (Aligianis et al., 2002; Kohl et al., 2002), *PDE6C* (cone cyclic GMP–specific 3′,5′ cyclic phosphodiesterase α; ACHM5, MIM 600827 and COD4, MIM 613093) (Chang et al., 2009; Thiadens et al., 2009), and *PDE6H* (cone cyclic GMP–specific phosphodiesterase γ; MIM 601190; ACHM6 or RCD3A, MIM 610024) (Kohl et al., 2012). Three in four patients carry mutations in *CNGA3* or *CNGB3*, the genes encoding the two subunits of the cyclic nucleotide-gated (CNG) channel in cone photoreceptors (Kohl et al., 2005; Poloschek and Kohl, 2010). The cone photoreceptor CNG channel is an essential component of the visual transduction cascade and translates light-dependent changes in the second messenger cyclic guanosine monophosphate (cGMP) into electrical activity, which in turn controls the release of neurotransmitters at the synapses to secondary neurons. The cone CNG channel forms a heterotetrameric complex composed of three CNGA3 and one CNGB3 subunits (Shuart et al., 2011). The absence of CNG channels in cone photoreceptor outer segments of ACHM patients results in complete lack of cone photoreceptor function.

We have previously shown that a genetic inactivation of *Cnga3* in mice—like in human patients—leads to selective loss of cone mediated light responses (Biel et al., 1999) accompanied by morphological, structural, and molecular changes, and finally results in cone cell death (Michalakis et al., 2005). The pathophysiological changes include a disorganization of cone membrane structure, mislocalization of cone opsins, down-regulation of outer segment proteins and degeneration of cones which progresses in a ventral to dorsal gradient with the fastest rate of degeneration in the ventral retina of *Cnga3* KO mice.

Currently, no treatment for ACHM is available, but several translational research programs aim at the development of curative treatments for ACHM (Michalakis et al., 2017). As described above, ACHM is a result of loss-of-function or missense mutations in certain genes and is inherited in an autosomal recessive manner. Therefore, gene supplementation therapy providing healthy copies of affected genes is a promising approach to cure ACHM. Over the past years, retinal application of AAVs has proven to be safe and efficient in various disease models (Schön et al., 2013; Petit et al., 2016; Michalakis et al., 2017). Current clinical trials include RPE65-linked LCA, choroideremia and achromatopsia (Petit et al., 2016; Scholl et al., 2016).

Focusing on *CNGA3*-linked ACHM, we have previously demonstrated restoration of visual function following adeno-associated virus (AAV) vector-mediated gene supplementation therapy in the *Cnga3* KO mouse model (Michalakis et al., 2010). The treatment resulted in reestablishment of regular CNG ion channel complexes in cone outer segments. In addition, normal cGMP levels were restored, which enabled the previously unresponsive cones to gain the ability to generate specific light responses.

Here, we provide novel data on further aspects of the therapeutic outcome beyond the initial proof-of-concept studies. Particular interest was given to intermediate and long-term follow-up of the treatment effect in *Cnga3* KO mice and the use of different AAV serotypes.

## Materials and methods

### Animals and ethics statement

Animals were housed under standard white cyclic light (200 lux, 12 h dark-light periods), had free access to food and water, and were used irrespective of gender. All procedures involving animals were performed in accordance with the ARVO Statement for the Use of Animals in Ophthalmic and Vision Research. The study and the protocol were approved by the competent local authority, Regierungspräsidium Tübingen, basing on the assessment by the appointed regional ethics board.

### Cloning and production of AAV vectors

Cloning and mutagenesis were performed by standard techniques. All sequence manipulations were confirmed by sequencing. Single-strand genome AAV2/5 Y719F (Michalakis et al., 2010) or AAV2/8 Y733F (Koch et al., 2012) pseudotyped AAV2 vectors expressing mouse *Cnga3* cDNA (Michalakis et al., 2010) under control of a 0.5 kb mouse blue (S) opsin (Michalakis et al., 2010) were produced as described recently (Becirovic et al., 2016). Physical titers (in vector genomes/μl) were determined by quantitative PCR on a LightCycler 480 (Roche Applied Science, Mannheim, Germany) using KAPPA SYBR FAST kit (Peqlab, Erlangen, Germany) and the following primer set: WPREF: 5′-AGTTGTGGCCCGTTGTCAGG-3′ and WPRER: 5′-AGTTCCGCCGTGGCAATAGG-3′.

### Subretinal AAV injections

A detailed protocol for optimized subretinal injection technique was described previously (Mühlfriedel et al., 2013). In brief, mice were anesthetized by subcutaneous injection of ketamine (66.7 mg/kg) and xylazine (11.7 mg/kg), and their pupils were dilated with tropicamide eye drops (Mydriaticum Stulln; Pharma Stulln, Germany). Young mice (postnatal 14 days) received a single subretinal injection of 1 μl of AAV particles (~5 × 10^9^ total vector genomes) at the ventral part, whereas in aged animals (from postnatal month one), due to the ventral-dorsal gradient of cone degeneration, the viral particles were applied at the dorsal part. The injection was performed free hand under a surgical microscope (Carl Zeiss, Germany) using a NanoFil injection kit (WPI, Germany) equipped with a 34 gauge beveled needle. In the experimental design, one eye was treated whereas the untreated eye served as the untouched control. Special care was taken to avoid damage of the lens. After injection, *in vivo* imaging was performed (described in next section). Animals were placed on a warming blanket and eye ointment (Vidisic®, Dr. Mann Pharma, Berlin, Germany) was applied to the corneas to prevent drying while the animals recovered from anesthesia. In addition, an antibiotic eye ointment was applied two times daily for 48 h (Dexamytrex®, Bausch & Lomb, Berlin, Germany).

### Qualitative analysis by *in vivo* imaging techniques

The quality of the injection and retinal morphology were examined immediately after subretinal injection using confocal scanning laser ophthalmoscopy (cSLO) and spectral domain optical coherence tomography (SD-OCT). The cSLO procedure was reported previously (Seeliger et al., 2005). For consecutive examinations at later time points, mice were anesthetized and pupils were dilated as described in section “subretinal AAV injections.” cSLO images were obtained with a Heidelberg Retina Angiograph (HRA I) at 10 and 20° field of view. Two different imaging modalities were used to visualize the retina: (1) 514 nm laser excitation, red free imaging mode, (2) and 830 nm laser excitation, infrared imaging mode. SD-OCTs were performed using a Spectralis TM HRA + OCT device from Heidelberg Engineering (Fischer et al., 2009; Huber et al., 2009). Imaging data were analyzed using the software package Eye Explorer version 3.2.1.0 from Heidelberg Engineering. Resulting data were exported as 24 bit color image files and processed in Adobe Photoshop CS3 (Adobe Systems, San Jose, CA, USA) (Garcia Garrido et al., 2015). If the procedure was not successful (severe damage like a full retinal detachment, lens opacity due to injury), the mice were excluded from further analysis.

### Functional analysis by Ganzfeld electroretinography

Electroretinograms (ERGs) were recorded according to the previously described procedures (Tanimoto et al., 2009). The ERG system consisted of a xenon light source, a Ganzfeld bowl, a signal amplification system (band-pass 0.3–300 Hz), a PC-based control and recording unit, and a monitor (Multiliner Vision; VIASYS Healthcare GmbH, Hoechberg, Germany). Animals were dark adapted overnight, anesthetized and pupils were dilated as described in section “subretinal AAV injections.” Single flash intensity series data were obtained under photopic (light-adapted 10 min at 30 cd/m^2^) conditions. Flash stimuli ranged from −2.0 to 1.5 log cd^*^s/m^2^ divided into ten steps. Responses to trains of brief flashes (flicker) of fixed luminance (1 log cd^*^s/m^2^) but varying frequency (0.5, 1, 2, 3, 5, 7, and 10 Hz) were recorded at the cornea under light-adapted conditions with a background illumination of 30 cd/m^2^. Flicker responses were averaged either 20 times (for 0.5–3 Hz) or 30 times (for 5–10 Hz). Single flash intensity and flicker frequency series data were obtained from treated animals (one treated eye, one untreated eye) at indicated time points after treatment.

### Immunohistochemical anaylsis

Vertical cryosections (10 μm) of the mouse retina were prepared for immunohistochemical staining as described previously (Michalakis et al., 2005). For an appropriate localization of the treated and untreated retinal part, eyes were marked temporally before harvesting. Antibodies used were rabbit anti-mouse CNGA3 polyclonal antibody (Michalakis et al., 2005, applied at 1:3,000 dilution) and rabbit anti-mouse S opsin polyclonal antibody (Millipore, applied at 1:300 dilution). For secondary detection donkey Cy3 anti-rabbit IgG was used (Jackson ImmunoResearch). Cone photoreceptors were stained with fluorescein isothiocyanate conjugated peanut agglutinin (FITC-PNA; 1:100, Sigma-Aldrich). Confocal images were collected on a LSM 510 (Carl Zeiss, Oberkochen, Germany) microscope.

### Whole mount stainings

Animals were sacrificed, eyes were enucleated and immediately fixed in buffered 4% paraformaldehyde for 10 min. For an appropriate localization of the treated and untreated retinal part, eyes were marked temporally before harvesting. For immunostaining, retinal whole mounts were washed with 0.1M PB (3 × 10 min) and co-stained with FITC-PNA and rabbit anti-mouse CNGA3 polyclonal antibody over night. For secondary detection, a donkey Cy3 anti-rabbit IgG was used. After washing with 0.1M PB the retinas were flat mounted on objective slides, orientated and embedded in mowiol mounting medium. Pictures were taken using a fluorescence microscope connected to a video camera (Zeiss ApoTome, AxiocamMRm, Germany).

### Statistical analysis

In this study, two different types of Student's *t*-tests were used for statistical analysis to determine if two sets of data are significantly different from each other. The independent samples *t*-test (unpaired independent samples) was used when two separate sets of independent and identically distributed samples were obtained, one from each of the two populations being compared were non-overlapping (e.g., when comparing the effects of the two capsid variants). The paired *t*-test (paired dependent samples) was used to when one group was tested on two time consecutive points (e.g., repeated measurements at 1 and 12 months PI).

## Results

### Subretinal delivery of AAV vectors and *in vivo* monitoring of the injection procedure

**Figure 1:**
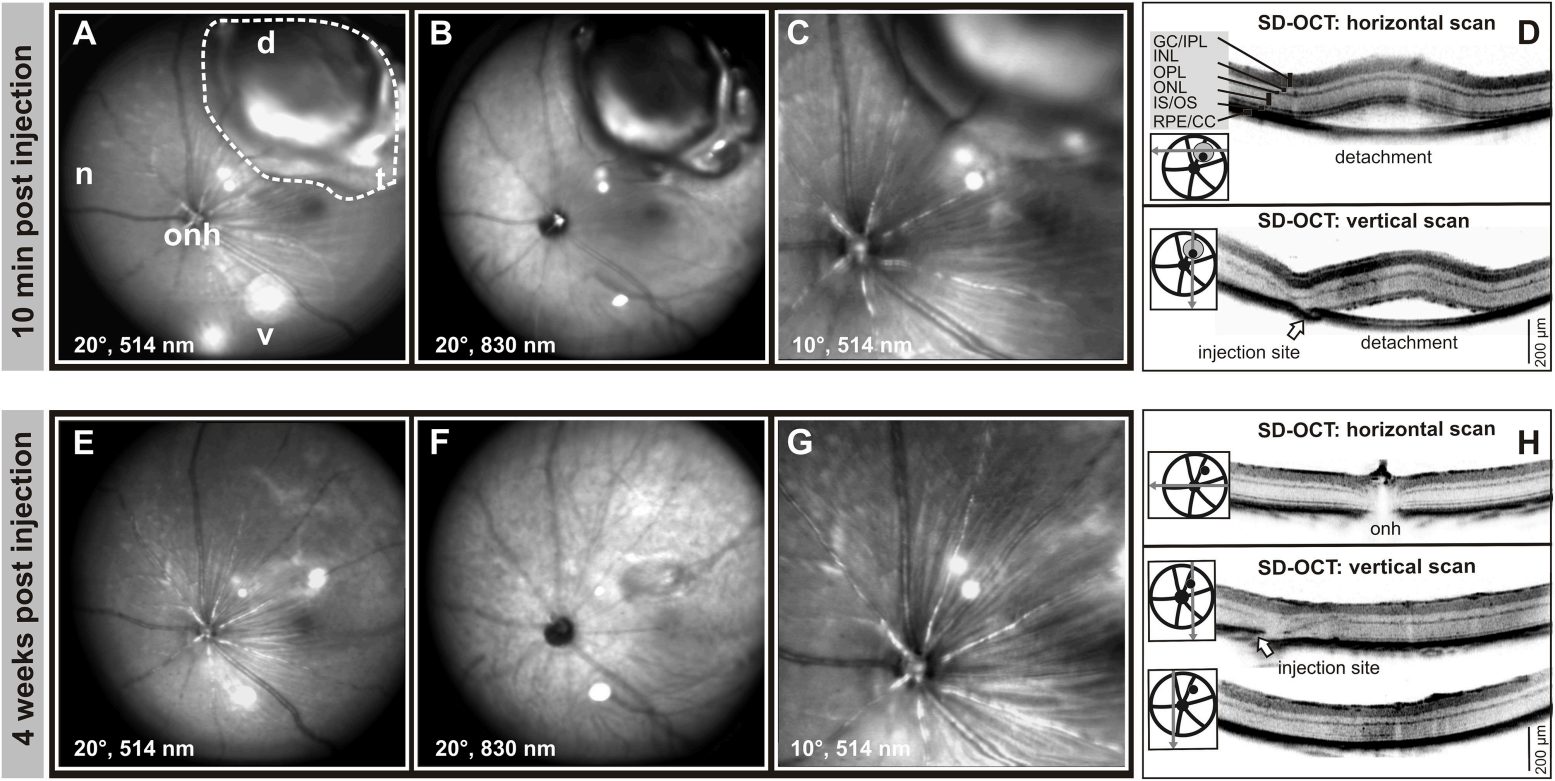
**Monitoring of retinal integrity after subretinal AAV delivery. (A–H)** *In vivo* cSLO and SD-OCT scans of the subretinal bleb 10 min **(A–D)** and 4 weeks **(E–H)** after injection at PM1. The dashed line indicates the area of the bleb due to subretinal detachment, arrows mark the injection site. A total volume of 1.0 μl AAV 2/8 (Y733F)-S opsin-Cnga3 was injected into the dorsal-temporal part of the retina. d, dorsal; n, nasal; t, temporal; v, ventral; onh, optic nerve head. GCL, ganglion cell layer; IPL, inner plexiform layer; INL, inner nuclear layer; OPL, outer plexiform layer; ONL, outer nuclear layer; IS, inner segments; OS, outer segments; RPE, retinal pigment epithelium; CC, choroid complex.

Each animal received a single subretinal injection of AAV vector in one eye, while the other eye remained untouched and served as control. To minimize any possible damage due to the surgical procedure a total volume of 1 μl AAV vector suspension was injected. The technical outcome was monitored via *in vivo* imaging of the mouse eye using confocal scanning-laser ophthalmoscopy (cSLO) and spectral domain optical coherence tomography (SD-OCT) within 10 min after subretinal delivery of the AAV vector (Figure 1). Following the injection of 1 μl vector suspension to the dorsal retina, a defined local subretinal bleb became evident in cSLO fundus imaging (Figures 1A,B). A higher magnification (10° field of view) revealed the trajectory of retinal blood vessels at the border of the transitory subretinal bleb (Figure 1C). Subsequent assessment of horizontal and vertical SD-OCT scans confirmed the detachment of the retina from the RPE at the subretinal bleb area (Figure 1D). This subretinal detachment resolved spontaneously within a few days, and after 4 weeks only subtle morphological abnormalities around the injection site and within the former detached area remained detectable (Figures 1E–H).

**Figure 2:**
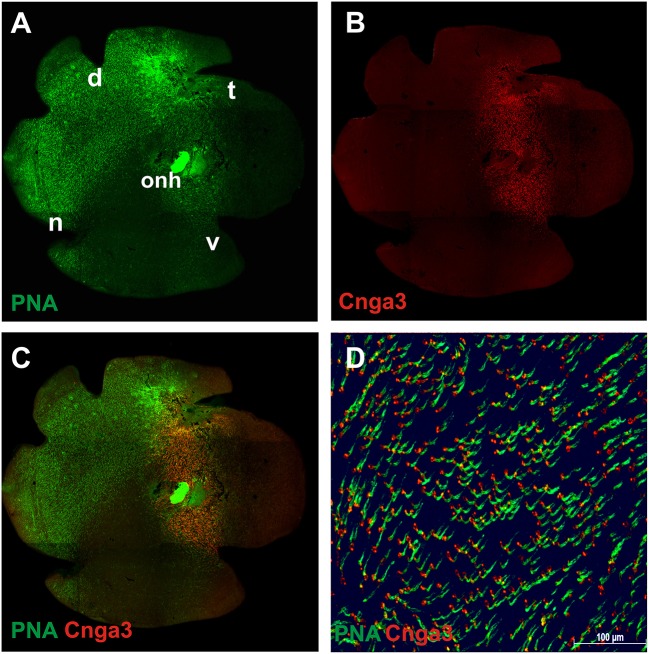
**Immunolabeling of flat-mounted treated ***Cnga3*** KO retina. (A–D)** Representative montage images of epifluorenscence imaging of a treated *Cnga3* KO retina mounted with the photoreceptor side up. The animal was treated at the age of 2 weeks with AAV2/8 (Y733F)-S opsin-Cnga3 and imaged at 4 months after treatment. **(A)** Cones were stained with the marker peanut agglutinin (PNA, green). In 4-month-old *Cnga3* KO mice, the majority of ventrally located cones, specifically of blue opsin expressing (S opsin) cones, are lost in untreated regions. **(B)** Immunosignal obtained with a Cnga3-specific antibody (red) revealed that about 1/3 of the treated *Cnga3* KO retina was transduced with a single injection and expressed the AAV vector-encoded Cnga3 protein. **(C)** Merged image showing an overlay of Cnga3 (red) and PNA (green) signals. **(D)** Magnified detail of the treated area as shown in **(C)**. d, dorsal; n, nasal; t, temporal; v, ventral; onh, optic nerve head.

To assess the extent of transduction obtained within the area of the subretinal injection, we harvested the retina from mice treated with a dose of ~5 × 10^9^ total vector genomes of AAV2/8 (Y733F)-S opsin-Cnga3, expressing murine *Cnga3* under control of the mouse S opsin promoter. After labeling with a specific anti-CNGA3 antibody and the cone photoreceptor marker peanut agglutinin (PNA), the retina was flat-mounted and imaged under an epifluorescence microscope (Figures 2A–D). As described earlier, the natural course of cone photoreceptor degeneration in this model follows a ventral to dorsal gradient (Michalakis et al., 2005). Consequently, at 4 months post-injection (PI), only a few remaining cones were labeled by the cone marker PNA in the ventral half (V) of the flat-mounted *Cnga3* KO retina (Figure 2A). Injections in this set of experiments were placed in the ventral part, however, transduction of the temporal or nasal parts of the retina due to vector diffusion could not be excluded. Immunolabeling confirmed the presence of mouse Cnga3 protein in the treated part of the retina surrounding the injection site (Figure 2B). The area of cone-specific expression of CNGA3 (red) following the single subretinal injection spanned about 1/3 of the entire retina. A closer inspection of the overlay of the Cnga3 expression pattern with the PNA staining revealed a substantial number of PNA-positive cones in the ventral half of the treated *Cnga3* KO retina (Figures 2C,D). As this area normally lacks cones at this stage of degeneration (Michalakis et al., 2005), one can conclude that the extended cone survival is related to the treatment.

### Therapeutic capacity of two different modified capsids AAV2/5 (Y719f) and AAV2/8 (Y733f)

**Figure 3:**
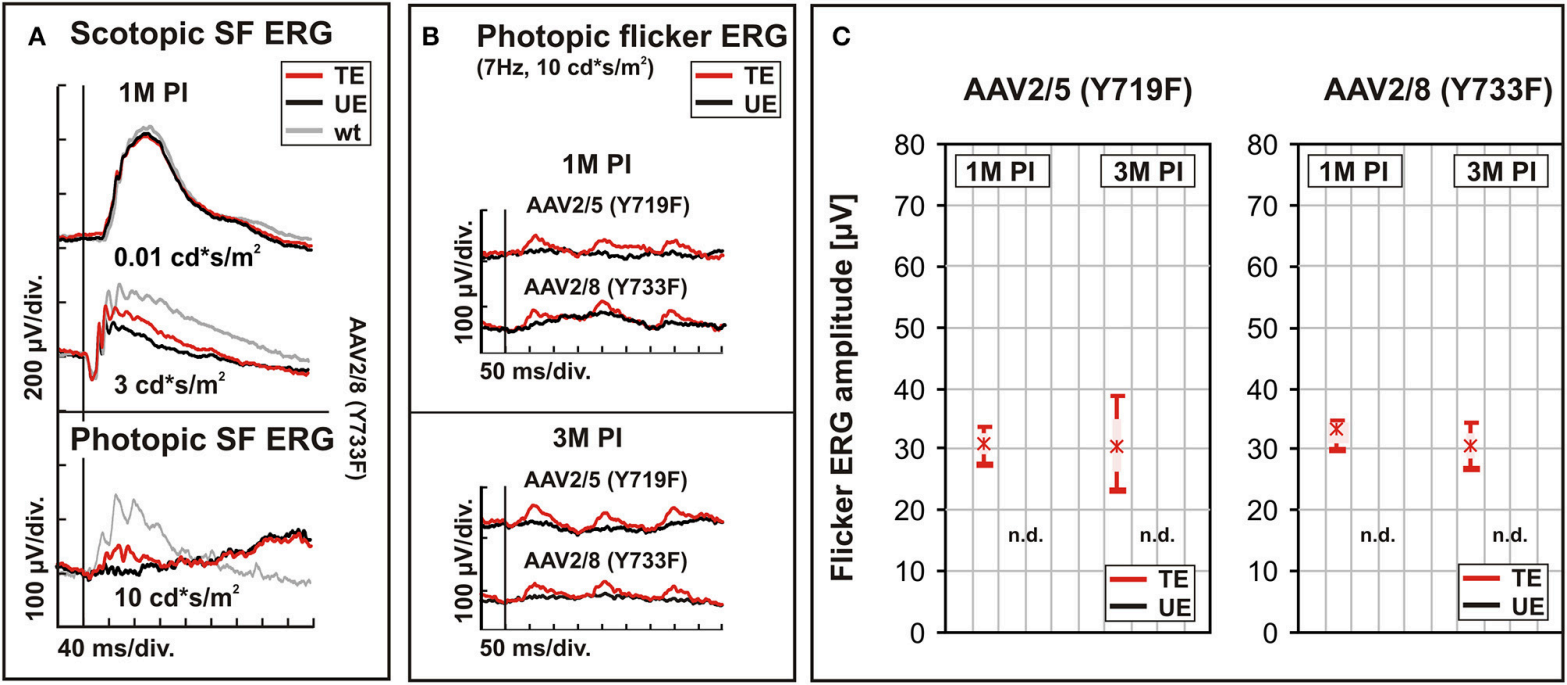
**Functional performances of AAV2/5 (Y719F) and AAV2/8 (Y733F) vector serotypes ***in vivo***. (A–C)** *Cnga3* KO mice were treated at an age of 2 weeks with AAV2/5 (Y719F)- or AAV2/8 (Y733F)-S opsin-Cnga3 vectors and the functional outcome was assessed at 1 and 3 months PI. **(A)** Representative single flash ERG traces obtained under scotopic (dark-adapted) or photopic (light-adapted) conditions. Top traces: scotopic rod system response, center traces: scotopic mixed rod/cone system response, bottom traces: photopic cone system response. **(B)** Comparison of photopic 7 Hz flicker ERG responses obtained from representative animals treated with AAV2/5 (Y719F) or AAV2/8 (Y733F) serotypes. Top panel: evaluation at PM1 PI, bottom panel: evaluation at PM3 PI. **(C)** Box plots summarizing the corresponding group data (*n* = 10). ERG b-wave amplitudes of TEs are shown in red and those of UEs in black. Boxes indicate the 25–75% quantile range, whiskers the 5 and 95% quantiles and solid lines connect the medians of the data. As no flicker waveform could be detected in any of the UEs, the result are indicated as “n.d.” (non-detectable). SF, single flash.

To evaluate potential differences in the therapeutic capacity of two different modified pseudotyped AAV2 vectors expressing mouse Cnga3 cDNA (AAV2/5 Y719F and AAV2/8 Y733F), we compared the outcome in two groups of respectively treated *Cnga3* KO mice for a period of up to 3 months PI. Retinal function was assessed via Ganzfeld ERG to investigate the contribution of the rod and the cone system (Figure 3). Figure 3A shows a comparison of wild-type (wt) mouse data to the ERGs of the AAV2/8 (Y733F)-treated and untreated eyes of a *Cnga3* KO animal. Under scotopic (dark-adapted) conditions, the specific rod system response (Figure 3A, top) revealed no difference between the treated eye (TE, red traces), untreated eye (UE, black traces), and the wild-type (wt) eye (gray traces). This finding indicates that the injection procedure had no measurable deleterious effects on retinal functionality, and corroborates the published data for AAV2/5 (Y719F) (Michalakis et al., 2010). Use of a more intense flash under scotopic conditions [ISCEV standard flash, Marmor et al., 2004], which potentially evokes mixed rod/cone system responses, revealed a higher b-wave amplitude in the TE relative to the UE, indicating a contribution of the cone system (Figure 3A, lower part of top graph). Cone function may usually best be observed under photopic, light adapted conditions, as in the bottom graph of Figure 3A. The respective photopic single flash recording clearly shows the treatment effect on cones in the TE, while there is merely a zero baseline in the UE. Again, these results in AAV2/8 (Y733F) corroborate the published data for AAV2/5 (Y719F) (Michalakis et al., 2010), including the fact that the approximate size of the treated area of 1/3 of the retina corresponds to an ERG amplitude of 1/3 to 1/4 of wt.

A particularly valuable biomarker for cone function is the photopic flicker ERG. For the direct comparison between AAV2/5 (Y719F) and AAV2/8 (Y733F), we chose a flash frequency of 7 Hz (Figure 3B). The presence of three consecutive flash responses in a trace enhances the visibility of differences and the detection of potential rescue effects. In general, Figure 3B again clearly shows the benefit of the intervention on cone functionality for both serotypes, and for both observation intervals at 1M and 3M post-injection (PI). Due to the complete lack of both cone (*Cnga3* KO) and rod responses (which were desensitized with background light), the UEs are unable to respond, while there are distinct responses in the TEs (Figure 3B). We found no visible differences in cone system functionality between the AAV2/5 (Y719F) and the AAV2/8 (Y733F) groups, neither in the waveforms (representative traces in Figure 3B) nor in the group amplitude data (box plots) summarizing the results at 1M and 3M PI for each serotype (Figure 3C). The traces of the UEs did not contain any discernible waveforms, so that no amplitudes were measurable, as marked by “n.d.” for “non-detectable” in Figure 3C. A statistical work-up of the data summarized in Figure 3C revealed

- no differences between AAV2/5 (Y719F) and AAV2/8 (Y733F) at 1M (*p* = 0.94) and 3M (*p* = 0.75),
- no differences between AAV2/5 (Y719F) at 1M and AAV2/5 (Y719F) at 3M (*p* = 0.21), and
- no differences between AAV2/8 (Y733F) at 1M and AAV2/8 (Y733F) at 3M (*p* = 0.64).

As (b) and (c) involve follow-up data from the same individual animals, a paired *t*-test was applicable, while an unpaired *t*-test was used in (a) as the data originate from different mice. These findings suggest that a robust rescue of the *Cnga3* KO phenotype was achieved over a 3 months period with both AAV vector serotypes, AAV2/5 (Y719F) and AAV2/8 (Y733F).

### Long-term performance of AAV-mediated *Cnga3* gene supplementation therapy

**Figure 4:**
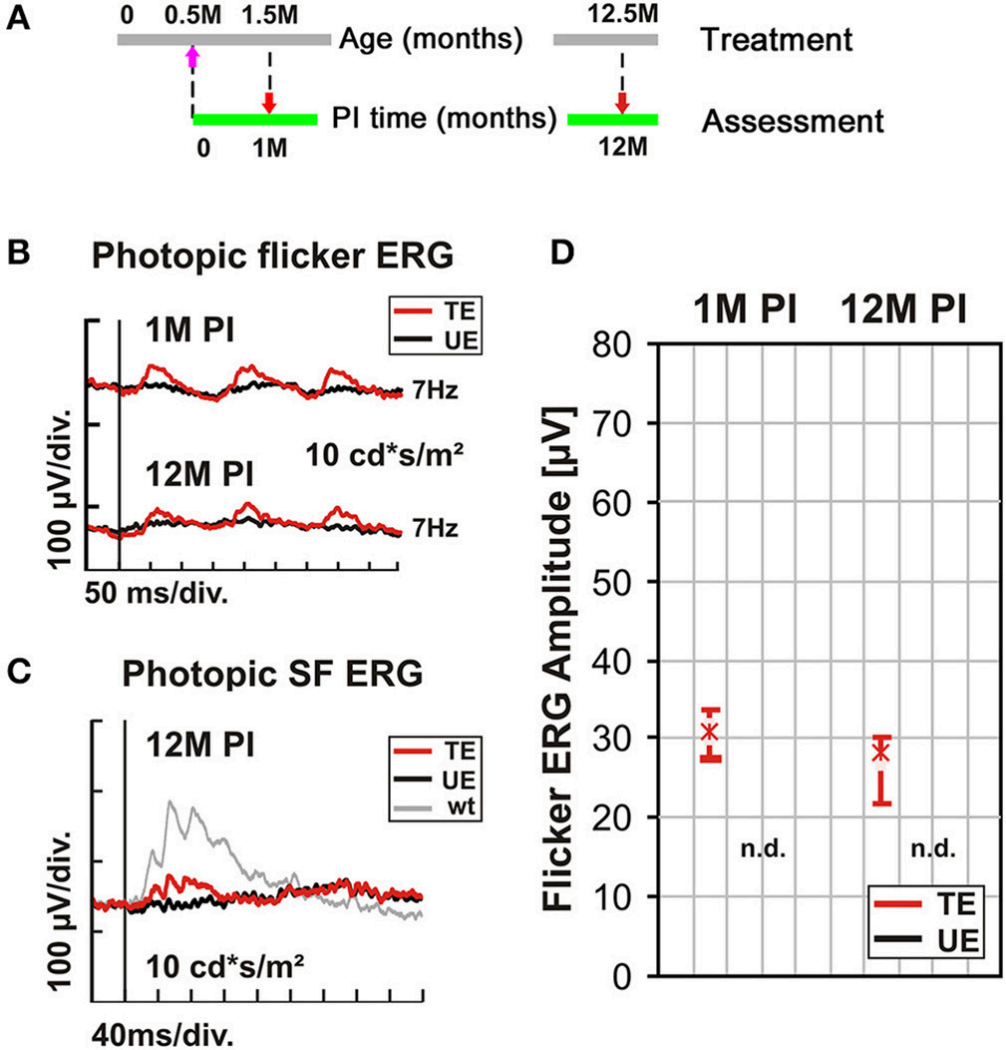
**Long-term outcome of AAV treatment. (A–D)** *Cnga3* KO mice were treated with AAV2/5 (Y719F)-S opsin-Cnga3 and analyzed according to the scheme depicted in **(A)**. **(B)** Representative photopic (light-adapted) 7 Hz flicker ERG traces obtained at 1M and 12M PI. **(C)** Photopic (light-adapted) single flash ERG recordings from a treated (TE, red trace), untreated (UE, black trace), and wild-type (wt) eye (gray trace) obtained at 12M PI. **(D)** Box plots summarizing the corresponding flicker ERG group data (*n* = 14). Flicker ERG amplitudes of TEs are shown in red and those of UEs in black. Boxes indicate the 25–75% quantile range, whiskers the 5 and 95% quantiles and solid lines connect the medians of the data. As no flicker waveform could be detected in any of the UEs, the result are indicated as “n.d.” (non-detectable). PM, post natal month; PI, post-injection. SF, single flash.

A key outcome of any gene supplementation therapy is a sustained, optimally life-long rescue. Published data (Michalakis et al., 2010) and the results shown above document a beneficial effect of the treatment up to 3 months PI. Here, we investigated the performance of the treatment over a period of 12 months PI, as the average lifespan of mice does not permit longer periods without unduly increasing the risk of systematic errors. In these long-term experiments, the AAV2/5 (Y719F)-S opsin-Cnga3 vector was used, and 1 μl subretinally injected into the ventral retina of 2-week old *Cnga3* KO mice. The functional assessment of cone photoreceptor function was done at 1 M and 12 M PI (Figure 4A). Figures 4B,C summarizes the Ganzfeld ERG recordings with a focus on the biomarker used in previous sections (7 Hz photopic flicker ERG). For comparison to Figure 3A, the results of the photopic single flash ERG are included in Figure 4C. As illustrated in Figures 4B,D, cone-mediated function persisted at a similar level over the entire observation period of 12 months. Consequently, a statistical analysis as described above based on the data summarized in Figure 4D indicated no significant differences in treatment effects (*p* = 0.5). This result is particularly encouraging as the treated area covered parts of the ventral retina where, without therapy, cones are completely lost after 4 months (Michalakis et al., 2005), indicating that these targeted cones were saved.

**Figure 5:**
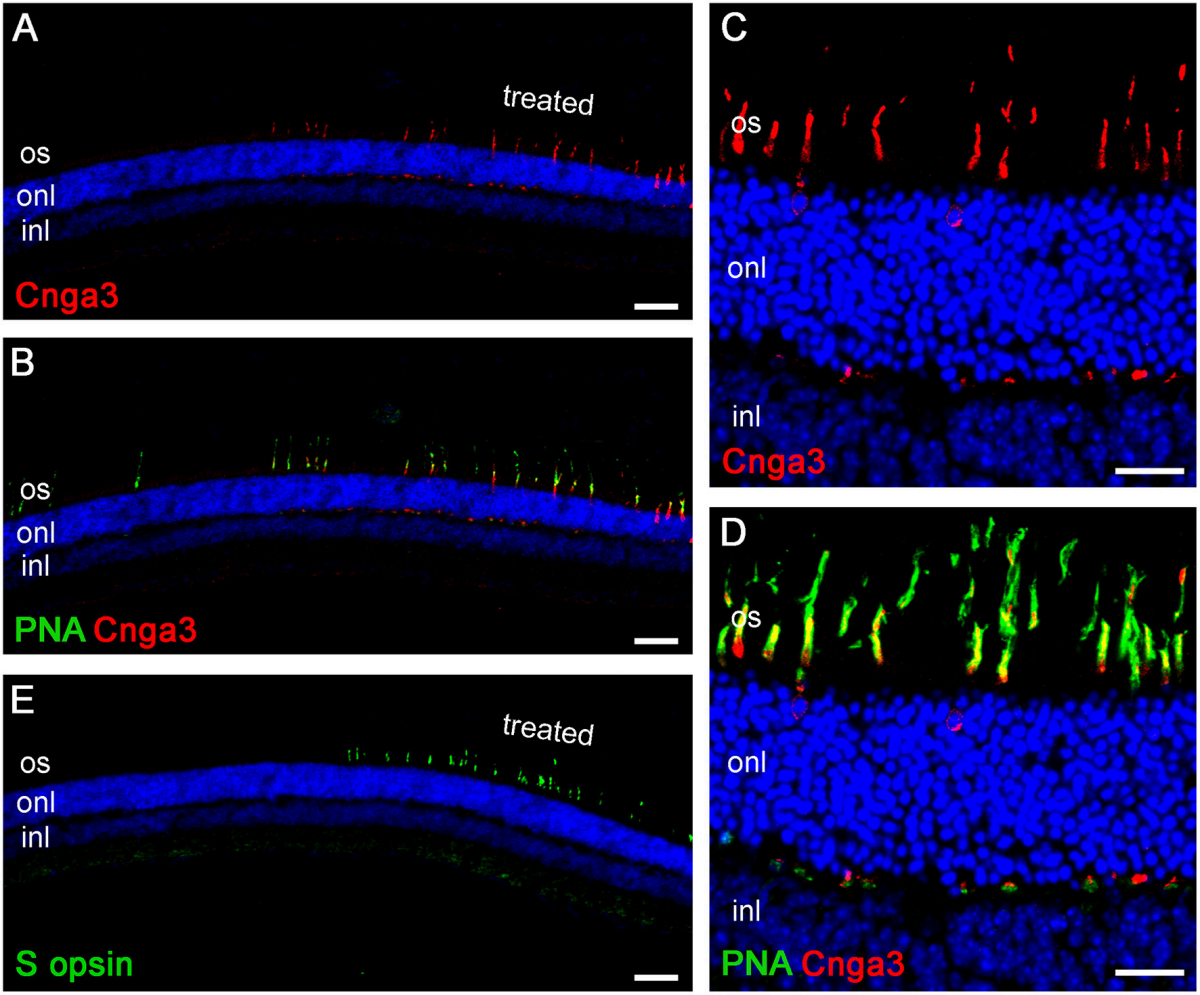
**Immunohistological evaluation of an AAV-treated ***Cnga3*** KO retina at 12M PI. (A–E)** Representative confocal scans of vertical cryo-sections obtained from the retina of *Cnga3* KO mice at 12 months (12M) after treatment with AAV2/5 (Y719F)-S opsin-Cnga3. Staining was performed using specific antibodies and the cone-specific marker peanut agglutinin (PNA). Cell nuclei were marked with Hoechst 33342. **(A–D)** Low magnification survey **(A,B)** and high magnification detail images **(C,D)** featuring Cnga3 immunosignal (red) alone **(A,C)** or in combination with the PNA signal (green) **(B,D)**. The region depicted in **(A,B)** spans from the treated (right) to the untreated (left) part of the retina. **(C,D)** shows higher magnification details from the treated part. **(E)** Overview image from a consecutive cryo-section to the section shown in **(A,B)** stained with an S opsin-specific antibody. The S opsin signal (green) indicates preservation of this subtype of cones at 12M PI.

Upon completion of the ERG measurements, the retinas were harvested for immunohistological analyses. We used the cone marker PNA and a CNGA3-specific antibody on vertical cryo-sections through the retina of treated *Cnga3* KO mice. A representative confocal overview image from a retinal section including a treated (right) and untreated (left) area is shown in Figure 5A. AAV vector-encoded CNGA3 protein is still clearly detectable at 12 M PI in cones within the treated, but not in the untreated part of the *Cnga3* KO retina (Figure 5A). These CNGA3-positive cones were also positive for the cone marker PNA (Figure 5B) whereas only a few surviving CNGA3-negative cones were found (see left, untreated part in Figure 5B). Higher magnification images show that most of the CNGA3 protein localized to cone outer segments, but some protein was also observed in cone cell bodies and synapses (Figures 5C,D). S opsin-positive cones are not found in untreated *Cnga3* KO retina older than 4 months of age (Michalakis et al., 2005). However, S opsin-positive cones still survived at 12 M PI in the treated ventral part of *Cnga3* KO retina (Figure 5E), suggesting that the treatment may halt degeneration of the fast degenerating S opsin-positive cones.

### Is the therapy still effective when applied at more advanced stages of disease?

**Figure 6:**
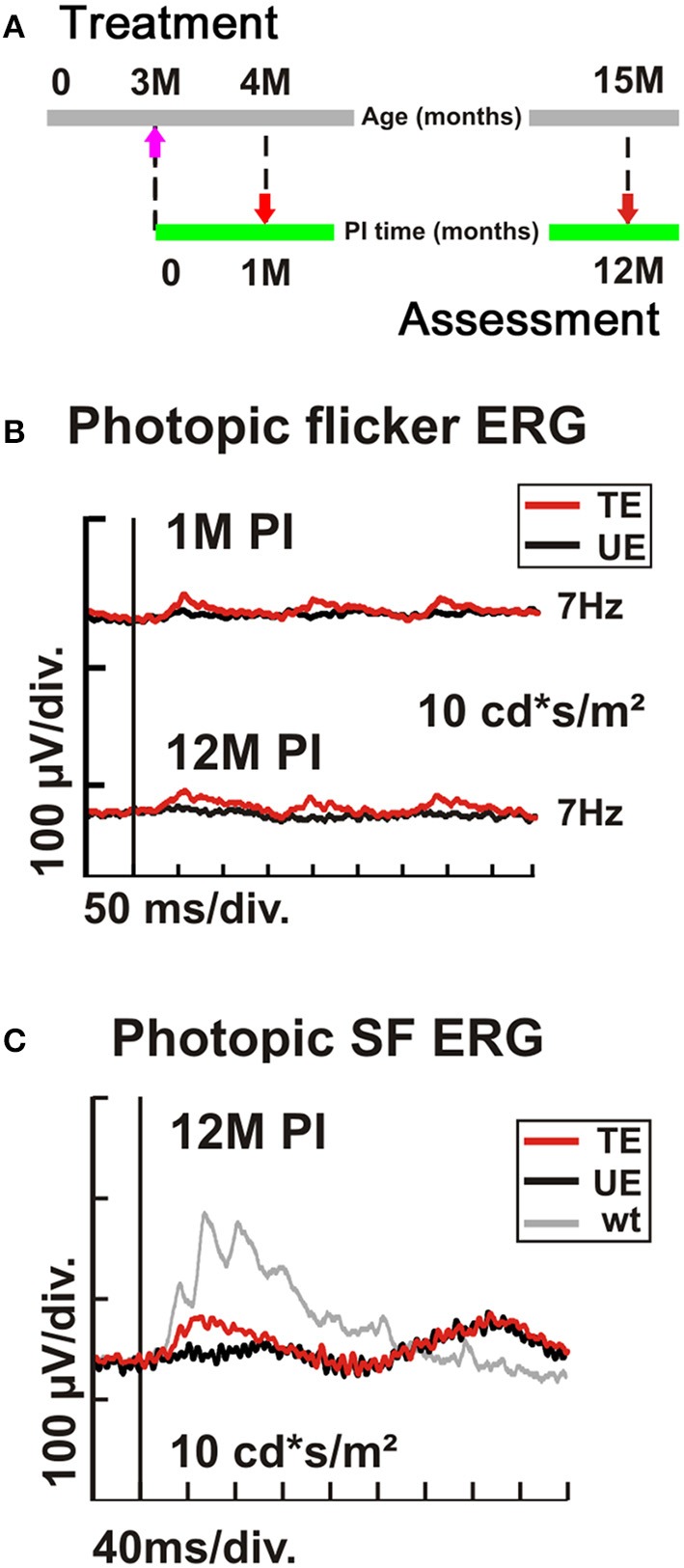
**Dependency of AAV treatment on age at intervention. (A–C)** *Cnga3* KO mice (*n* = 4) were treated with AAV2/8 (Y733F)-S opsin-Cnga3 and analyzed according to the scheme depicted in **(A)**. **(B)** Representative photopic (light-adapted) 7 Hz flicker ERG traces obtained at 1M and 12M PI when treated at PM3. **(C)** Photopic (light-adapted) single flash ERG recordings from a PM3-treated (TE, red trace), untreated (UE, black trace), and wild-type (wt) eye (gray trace) obtained at 12M PI. PM, postnatal month; PI, post-injection. SF, single flash.

Finally, we treated 3-month-old (PM3) *Cnga3* KO mice in the same way as before the 2-week-old animals, using the AAV2/8 (Y733F)-S opsin-Cnga3 vector (Figure 6A). Since the peak of cone cell death is at about postnatal day 35 (Arango-Gonzalez et al., 2014) and cone degeneration is particular present in the ventral *Cnga3* KO retina, we aimed at delivering the vector entirely to the dorsal retina to target the slower degenerating dorsal cones. We found that although presumably a substantial number of cones has already been lost at time of treatment, it was still possible to endow the remaining cones with the ability to generate specific light responses (Figure 6B). The treatment effect persisted up to 12 M PI (Figure 6B). The functional benefit was confirmed by single flash ERG recordings (Figure 6C). In summary, the preclinical results in Cnga3 KO mice show that even at advanced stages of degeneration up to at least PM3, surviving cones are still treatable.

## Discussion

Achromatopsia is a severely disabling inherited retinal disease with a high clinical and socioeconomic importance. An estimated 80% of ACHM cases are caused by mutations in one of the two genes encoding the cone CNG channel (*CNGA3* and *CNGB3*). In Europe and the United States, *CNGB3* mutations are found in ~50% of patients, while *CNGA3* mutations are found in about 30% of cases (Kohl et al., 2005). Mutations in all other known ACHM genes together account for less than 6% of patients. With the discovery of genetic causes of human diseases, the creation of homologous animal models and the development of efficient gene transfer strategies for these previously non-treatable disorders became feasible. So far, several groups have focused on developing gene therapies for *CNGA3* or *CNGB3* (Michalakis et al., 2017). This includes a number of pivotal proof-of-concept studies for *CNGA3* and *CNGB3* gene supplementation in mouse models (Michalakis et al., 2010; Carvalho et al., 2011; Pang et al., 2012), dog models (Komaromy et al., 2010, 2013), or sheep models of ACHM (Banin et al., 2015). All studies utilized AAV vectors and indicated that supplementation of the missing gene product was able to rescue the ACHM phenotype. A particular asset of therapy in ACHM is that it enables previously non-functional cone photoreceptors to generate specific light responses reminiscent of normal cone responses. These encouraging results triggered the initiation of translational projects aiming at the transfer of such AAV-based gene supplementation treatments for ACHM into the clinics (Michalakis et al., 2017). However, the challenges of human gene supplementation therapy are multifaceted. They comprise the necessity to fully understand the etiopathology of the targeted disease and its genetic mechanisms.

In the present study, three important facts in the murine *Cnga3* gene supplementation became apparent:

- The functional restoration of the deficient murine cone system was independent of the two AAV serotypes used, AAV2/5 (Y719F) and AAV2/8 (Y733F). This is an important finding due to the fact that each AAV serotype shows unique transduction characteristics (Sanlioglu et al., 2001). In particular, the AAV serotypes' ability to transduce cones has been evaluated in the pig (Manfredi et al., 2013) and non-human primate (Vandenberghe et al., 2013) retinas, which present cone density and distribution more similar to humans than mice. Among these, serotypes AAV2/5 (Alexander et al., 2007; Mancuso et al., 2009; Komaromy et al., 2010), AAV2/8 (Manfredi et al., 2013), and AAV2/9 (Vandenberghe et al., 2013) were found to present the highest cone transduction properties. In addition to the large portfolio of naturally-occurring AAV serotypes, novel AAV capsid variants with enhanced gene transfer efficiency and altered tropism have been generated in recent years (Vandenberghe et al., 2009). Phosphorylation of specific tyrosine residues within the AAV capsid is thought to be responsible for AAV targeting to proteasome and degradation (Zhong et al., 2008). Therefore, the exchange of conserved tyrosine residues with phenylalanine in AAV capsids of different serotypes results in higher levels of transduction compared to wild-type AAV capsids, presumably allowing AAV particles to escape the proteasome and reach the nucleus. In particular, AAV2 (Y444F), AAV2 (Y730F), AAV8 (Y733F), and AAV9 (Y446F) have an efficacy similar to that of wild-type AAV at lower doses, a broader tropism within the neuronal retina and enhanced diffusion across the retina (Petrs-Silva et al., 2009). Our data reveal the efficacy of the treatment of Cnga3-deficiency by using two modified capsids, AAV2/5 (Y719F) and AAV2/8 (Y733F) thereby confirming these findings as described in the literature. While we describe here, for the first time, a stable treatment effect using the AAV2/5 (Y719F) variant, the AAV2/8 (Y733F) variant has been successfully used to drive long-term expression of PDE6B and improvement of the retinal phenotype in rd10 mice, a model of autosomal recessive RP, which is challenging due to the aggressive and severe PR degeneration (Pang et al., 2011).
- The general aim of viral vector-mediated gene supplementation therapy of inherited disorders is to provide long-term beneficial effects optimally after a single treatment. Therefore, it is always critical to assess the duration of the treatment effect in a relevant model. For *CNGB3*-linked ACHM long-term follow-up studies (up to 42 months) after Cngb3 supplementation in the *Cngb3*-deficient dog model have been published (Komaromy et al., 2010, 2013). For *CNGA3*-linked ACHM such long-term data were missing so far. Here, we show for the first time that AAV-mediated *Cnga3* gene supplementation in young treated *Cnga3* KO mice results in long-term, presumably lifelong beneficial effects on cone-mediated function for at least 12 months after treatment. At late time points beyond 12 months of age in mice, the treatment effect may become diminished due to natural aging. Because of the shorter life span and different metabolism of mice (Dutta and Sengupta, 2016), this range corresponds to a considerably high age in humans. Since we were lacking a cohort of wild-type mice tested under the same conditions and in the same intervals, the generation of a reference baseline for natural aging remains to be established in future work.
- As on one hand many hereditary eye diseases like RP are often diagnosed at advanced disease stages or on the other hand the pathology in some forms develops already before birth or very early in life, a curative gene therapy approach may come too late to efficiently tackle the degeneration process. Thus, it is of paramount importance to ascertain the therapeutic time window for efficient gene delivery in each disease. Here, we show that 3-month-old *Cnga3* KO mice may still be successfully treated. We were further able to confirm that this effect persisted for at least 12 months post-injection. This is remarkably given that the absolute age of the treated *Cnga3* KO mice in this case was 15 months and significant cell death and loss of cone photoreceptors can be observed in the *Cnga3* KO retina already at the age of 3 months (Michalakis et al., 2005; Arango-Gonzalez et al., 2014). At these advanced stages of the disease cone photoreceptors are barely detectable in the ventral half of the untreated *Cnga3* KO retina (see Figure 2 and Michalakis et al., 2005, 2010; Arango-Gonzalez et al., 2014). Importantly, the finding that we were still able to rescue the ACHM phenotype in 3-month-old *Cnga3* KO mice suggest a rather wide therapeutic time window of opportunity.

In conclusion, we found that AAV-mediated Cnga3 gene supplementation therapy was effective with both AAV2/5 (Y719F) and AAV2/8 (Y733F) capsid serotypes in *Cnga3* KO mice. Our long-term data indicate a persistence of the treatment effect for an observational period of 12M PI even when applied at an advanced stage of cone degeneration at least up to PM3. These results hold promise for respective clinical trials in ACHM patients (e.g., clinical trials.gov identifiers: NCT02610582, NCT02599922, and NCT03001310). Given that mice lack a macula it is hard to extrapolate the present data and predict the outcome of clinical studies on ACHM. However, based on our findings, we can assume that even small, cone-rich areas in the macula are a valuable target for gene supplementation. Further studies have to clarify how many cones have to persist for a successful therapy. Our findings further suggest that sizable time windows for the efficient application of gene therapy may be available in a broader spectrum of gene-therapeutic approaches of hereditary eye diseases.

## Author contributions

RM: substantial contribution to conception and design of experiments, acquisition, analysis and interpretation of data drafting the work and final approval. SM: substantial contribution to conception and design of experiments, acquisition, analysis and interpretation of data, drafting the work and final approval. NT: acquisition, analysis of data, revising data for important intellectual content, drafting the work. CS: acquisition, analysis of data, revising data for important intellectual content, drafting the work. VS: acquisition and interpretation of data, revising data for important intellectual content, drafting the work. MG: acquisition and interpretation of data, revising data for important intellectual content, drafting the work. SB: acquisition of data, revising data for important intellectual content, drafting the work. GH: acquisition of data, revising data for important intellectual content, drafting the work. MB: substantial contribution to conception and design of experiments, interpretation of data, drafting the work. MS: substantial contribution to conception and design of experiments, interpretation of data, drafting the work and final approval.

### Conflict of interest statement

The handling Editor and author SM declare that they are co-hosting the Research Topic “Retinal Gene Therapy: From Basic Research to Translational Studies” but have no other collaborations. The other authors declare that the research was conducted in the absence of any commercial or financial relationships that could be construed as a potential conflict of interest.
